# Putting epigenetic biomarkers to the test for clinical trials

**DOI:** 10.7554/eLife.58592

**Published:** 2020-06-09

**Authors:** Jamie N Justice, Stephen B Kritchevsky

**Affiliations:** 1 Internal Medicine Section on Gerontology and Geriatrics, Wake Forest School of MedicineWinston-SalemUnited States; 2 Sticht Center for Healthy Aging and Alzheimer’s Prevention, Wake Forest School of MedicineWinston-SalemUnited States

**Keywords:** aging, biological aging, DNA methylation, epigenetic, human, life-course, Human

## Abstract

Reliable biomarkers are needed to test the effectiveness of interventions intended to improve health and extend lifespan.

**Related research article** Belsky DWW, Caspi A, Arseneault L, Baccarelli A, Corcoran DL, Gao X, Hannon E, Harrington HL, Rasmussen LJH, Houts R, Huffman K, Kraus WE, Kwon D, Mill J, Pieper CF, Prinz JA, Poulton R, Schwartz J, Sugden K, Vokonas P, Williams BS, Moffitt TE. 2020. Quantification of the pace of biological aging in humans through a blood test, the DunedinPoAm DNA methylation algorithm. *eLife* **9**:e54870. doi: 10.7554/eLife.54870

Geroscience is a developing discipline based on the premise that health can be improved by targeting aging. This hypothesis is supported by evidence that interventions (such as changes in diet) can improve the health and extend the lifespan of various animal models (López-Otín et al., 2013). Clinical trials are underway to test the geroscience hypothesis in humans (Barzilai et al., 2016). Definitive tests of the hypothesis must demonstrate reduced rates of age-related diseases and death, but the length of time and size of trial needed to test the hypothesis are both substantial. Therefore, objective, quantifiable characteristics of the aging process – known as biomarkers – that can be tracked in clinical trials are needed for the field to progress.

Useful biomarkers should meet several criteria: i) their measurement should be reliable and feasible; ii) they should be relevant to aging; iii) they should robustly and consistently predict trial endpoints, such as functional ability, disease, or death; and iv) they should be responsive to interventions such as treatments targeting aging biology (Justice et al., 2018). Practically speaking, this means that a change in the level of a biomarker should parallel changes in the susceptibility to disease, age of death, or loss of function. Interventions that target aging and support the geroscience hypothesis should therefore also lead to changes in these biomarkers, which will be reflected in the incidence or severity of age-related diseases and functional decline.

Biomarkers based on DNA methylation levels look promising. Briefly, these biomarkers quantify the proportion of cells in which a gene locus is methylated. Small but consistent changes in the methylation of some loci occur in organisms with older ages, and early methods for estimating age using epigenetics took advantage of these chronologic changes (Hannum et al., 2013; Horvath, 2013). However, critics argue that while these ‘clocks’ may be associated with chronological age, it is uncertain whether they reflect meaningful change in the context of interventions affecting the underlying biology.

Estimators based on the levels of DNA methylation are now being developed to detect a myriad of disease states and predict mortality and adverse health events, and each is unique to its calibration method. A few of these estimators are calibrated to detect aging-related outcomes, which makes them attractive as possible biomarkers for clinical trials in geroscience. Now, in eLife, Daniel Belsky (Columbia University) and colleagues in the United States, the United Kingdom, Denmark and New Zealand report the development of a new epigenetic biomarker called Dunedin Pace of Aging methylation (DunedinPoAm) that is able to detect how aging phenotypes change over time (Belsky et al., 2020).

The new biomarker relies on a composite measure called the Pace of Aging that was developed by Belsky and colleagues several years ago (Belsky et al., 2015). The Pace of Aging is calculated based on a number of age-related phenotypic changes that occur over time. In the new work this measure was used to calibrate and validate a DNA-wide methylation clock in four independent cohorts. This is in contrast to previous approaches in which methylation biomarkers were calibrated using endpoints such as chronological age, death, environmental exposure or other biomarkers.

Is DunedinPoAm developed to the point where it could be relied upon as a biomarker for clinical trials targeting biological aging? Figure 1 shows four criteria that are used to evaluate DNA methylation as a biomarker. DunedinPoAm appears to satisfy the first three criteria. It remains to be seen if it can satisfy the fourth, which involves being responsive to interventions. One of the cohorts used to validate the new approach consisted of middle-aged, non-obese adults enrolled in the CALERIE trial. This trial tested the effects of caloric restriction – an intervention that has been successful in animal models – over a period of two years. DunedinPoAm was able to predict changes in the Pace of Aging measure in the control group, but not in the group that had been calorie restricted. However, it remains to be seen whether interventions which affect aging biology change DunedinPoAm in a way that is consistent with the phenotypic changes observed in the trial.

**Figure 1.**
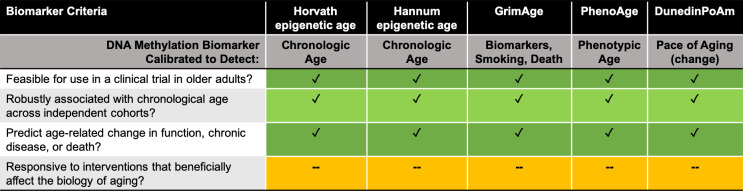
Assessing DNA methylation biomarkers for use in clinical trials in geroscience. How do five DNA methylation-based biomarkers (listed in row 1) fare when assessed against four biomarker criteria for clinical trials targeting aging (listed in column 1)? All five biomarkers meet the first three criteria, but none as yet satisfy the fourth criterion. Row 2 lists how each biomarker was calibrated. Horvath and Hannum are both epigenetic age estimators (Horvath, 2013; Hannum et al., 2013); GrimAge is a mortality estimator (Lu et al., 2019); PhenoAge is a health and lifespan biomarker (Levine et al., 2018); and DunedinPoAm is an estimator of change in aging phenotype over time (Belsky et al., 2020).

Testing the geroscience hypothesis in clinical trials is still in its early days, so it is not surprising that DunedinPoAm does not yet meet the primary criterion for an aging biomarker. However, emerging evidence suggests that methylation state may change with intervention. Data from two small clinical studies, with fewer than 15 people in the control and intervention groups, suggest that methylation-based biomarkers just might meet the minimum burden of proof (Chen et al., 2019; Fahy et al., 2019). However, the acid test for any biomarker will be whether changes in its levels predict differences in the rate of chronic disease accumulation or progression, death or other clinical trial endpoints. This will require a large study like the planned Targeted Aging with MEtformin (TAME) trial, which will last for over four years and include 3000 test subjects. This trial will test the effects of metformin, a drug currently used to treat type 2 diabetes, on FDA-informed clinical disease endpoints and functional ability. Trials like this will provide a platform for discovery, data sharing, and widescale biomarker validation to accelerate the pace of progress in geroscience.
